# A Treatise on Different Laborious Deliveries and on Polypi of the Uterus; with a Description of a New Lever, Constructed on the Plan of Roonhuysen's, and Compared with the Forceps; and Likewise of a New Instrument for the Ligature of Polypi, Approved by the Royal Academy of Surgery at Paris

**Published:** 1782

**Authors:** 


V. *Traité ſur divers accouchemens laborieux, et ſur les polypes de la matrice; ouvrage dans lequel on trouve la deſcription d'un nouveau levier, imité de celui de Roonhuyſen, et mis en parallele avec le forceps; ainſi que d'un nouvel inſtrument, propre à la ligature des polypes, approuvé par l'Academie Royale de Chirurgie de Paris. Par M.* G. Herbiniaux, *Chirurgien accoucheur et Lithotomiſte, à Bruxelles.* i. e. *A treatiſe on different laborious deliveries and on Polypi of the Uterus; with a deſcription of a new lever, conſtructed on the plan of Roonhuyſen's, and compared with the forceps; and likewiſe of a new inſtrument for the ligature of Polypi, approved by the Royal Academy of Surgery at Paris. By* G. Herbiniaux, *Surgeon Man-midwife and Lithotomiſt at Bruſſells.* 8vo. Bruſſells 1782. Vol. I. 430 pages with 3 copper-plates. Vol. II. 192 pages, with 1 copper-plate.

THE celebrated invention of Roonhuyſen to aſſiſt the delivery of the fœtus when the head is detained at the brim of the pelvis, which was ſo profitable to the inventor, and to thoſe who immediately participated of the ſecret from him or his ſucceſſors for near a century, ſeemed to loſe all its reputation, except in Hol-

 land, land, as foon as a defcription of it was communicated to the world by doctors Vifcher and Van de Poll. Thefe two phyficians, who practifed at Amfterdam, purchafed the fecret of the heirs of John de Bruyn, a furgeon of that city, for a fum equal to about 200 l. fterling, and publifhed it in the year 1753, together with fome papers of de Bruyn's defcribing the manner of ufing it.

Whether the coolnefs with which this difcovery was received by the public, was owing to the extreme fimplicity of the inftrument which confifted of a fingle piece of iron, a little curved at each end, or to the eagernefs with which the moft celebrated profeffors of midwifery were engaged in bringing to perfection the forceps, which had been invented about the fame time as the lever, and for the fame purpofe, is not eafy to determine. It is certain, however, that the critique which M. Levret publifhed on this inftrument contributed not a little to prevent it from being adopted in France, and it is probable that the filence of Dr. Smellie on this fubject had a fimilar effect in this country.

Profeffor Camper in a late publication (*Mem. de l'Acad. de Chirurgie*, tom. V.) has attempted to revive the credit of this inftrument, but fince the the appearance of his eſſay ſeveral works have been printed in France by meſſieurs Deleurye, Hoin, Baudelocque, and other pupils of the late M. Levret, in which the merit of the lever is not only placed far below that of the forceps, but the miſchiefs produced by the former ſeem to be exaggerated. The author of the work before us has undertaken to reply to the ſtrictures of thoſe writers. The whole of his firſt volume is employed on the lever.

After premiſing a ſhort hiſtory of the inſtrument, he begins with combating the criticiſms of M. Levret, which he aſcribes to motives of jealouſy. "I was not the only one—ſays M. Herbiniaux—who diſcovered the ſource of M. "Levret's cenſures; many of the pupils who "attended his lectures with me at the time the "deſcription of the lever was publiſhed, ob-"ſerved the effect it had upon that gentleman, "whoſe forceps had juſt begun to open to him "the way to fortune." After giving a long extract from M. Levret's *Suite des obſervations ſur les acccouchemens laborieux*, our author attempts to prove that Mr. L. was unacquainted with the true dimenſions and figure of the inſtrument, as well as with the manner of uſing it.

We are next preſented with the whole of Dr.

Camper's paper on this ſubject from the memoirs of the academy of ſurgery, accompanied with a critique to prove that the learned profeſſor was unacquainted with the manner of applying the inſtrument. This is alſo confirmed by a letter from M. Titſing (upon whoſe authority Dr. Camper had founded his relation of the manual) to our author: "I was very much "aſtoniſhed—ſays M. Titſing—to find this ce-"lebrated phyſician of opinion that the inſtru-"ment, when operating, is placed along the "ſide of the head, over the ear of the child, "with its point reſting upon the chin; and my "ſurprize was ſtill greater to ſee this ſpoken of "as my manner of uſing it, for I had always "found my inſtrument placed obliquely upon "the head, with its extremity fixed near the "maſtoid proceſs of the occipital bone." But although M. Titſing detects Dr. Camper's error in the manual of the lever, he does not contradict his account of the ſucceſs attending the uſe of it.

Meſſieurs Deleurye, Hoin, and Baudelocque have fallen into nearly the ſame miſtakes as Dr. Camper in the manual of the inſtrument, and were beſides unacquainted with the true dimenſions and figure of that uſed by the Dutch accoucheurs, coucheurs, for which they are ſeverely cenſured by our author.

M. Herbiniaux claims the merit not only of being the firſt perſon who diſcovered the intention of Roonhuyſen in making two curves, one larger than the other, to his inſtrument, but likewiſe that of having aſcertained the method of fixing the band which was obſerved round one of the ends of it when purchaſed of the heirs of de Bruyn. The deſcription of the inſtrument, as improved by himſelf, with directions for applying it, and ſome obſervations on the cauſes of laborious births, conclude the volume.

The lever of Roonhuyſen is deſcribed as a piece of iron 10$\frac{3}{4}$ inches long, one inch broad, and about $\frac{1}{8}$ of an inch thick. This iron is ſtrait in the middle for about four inches, but curved at each end; the curve at one extremity being an inch longer than the other. The longeſt curve is five-tenths of an inch deep, the ſhorteſt only one. The edges are poliſhed and rounded, but particularly at its extremities, which at the time of uſing the inſtrument were commonly covered with a plaſter. A ſtrong cord was fixed to the end end that was made uſe of, where the curve commenced.

Although it had been obſerved that the curves at the two extremities were of different ſizes, M. Herbiniaux was the firſt who after a variety of experiments found out the utility of this difference, and that it was eſſential to the perfection of the inſtrument, the larger curve being applicable only where the whole of the baſis of the ſcull is above the brim of the Pelvis. The want of attending to this diſtinction has been the cauſe, he thinks, of the little ſucceſs the French accoucheurs have had in uſing the inſtrument, and the conſequent contempt it has fallen into among them. They have uſually made it with a ſingle curve, and have adopted as their model, the largeſt, which they have alſo increaſed: this, our author contends, deſtroys its property as a lever, and brings it to the ſtate of a blunt crotchet.

M. Levret was the firſt who ſuggeſted the uſe of the cord fixed to the beginning of the curve, in order to draw downward with one hand, while with the other the handle of the inſtrument was carried upwards towards the belly of the mother: by this means not only the lever was prevented from ſlipping off the head of the fœtus, but but the preſſure upon the *ſymphyſis Pubis*, where it turns as upon an axis, was moderated.

This mechaniſm M. Herbiniaux has improved by making that part of the lever ſtronger, ſo as to admit of a hole for the ſtring to paſs through, by which means the cord, which is placed nearly under the arch of the Pubis, is prevented from galling the Vagina.

There are other alterations or amendments made by M. Herbiniaux in the inſtrument, which although not eſſential, contribute, he thinks, ſomewhat to the ſucceſs of the operation. His lever is made of ſilver, which he obſerves appears leſs terrible to the woman than one of iron. The inner ſurface of the curves is lined with unpoliſhed iron, which is leſs likely to ſlip from the head of the child than ſilver. For the ſake of rendering it more portable, it is divided into parts which ſcrew together when uſed, one of the curves only being mounted at one time. The handle is made round that it may be more eaſily held, and hollow to contain a ſyringe, by means of which the child may be baptized when that ceremony is thought neceſſary. The latter will not be conſidered as a very material improvement in this country.—

In

In the work itſelf the deſcription is illuſtrated by accurate engravings.

In making uſe of the lever, we are directed firſt to introduce a hand, or as much of it as we can into the vagina to diſcover the ſituation of the head. If the face is found turned a little obliquely to one ſide of the Pelvis, which is the moſt uſual ſituation, it is then, we are told, in the moſt favourable poſture for the application of the lever. Two fingers are now to be introduced between the ſymphyſis of the Pubis and the head of the child, or as near that part as may be, and one of the curved ends of the inſtrument is then to be inſinuated under the fingers, with its concave ſurface turned to the child, and moved a little backwards and forwards till its extremity meets with a proper degree of reſiſtance, which it will do as ſoon as it approaches the maſtoid proceſs of the occipital bone. The inſtrument being thus fixed we are to wait till a ſlight pain commences, and then the handle of the lever is to be carried upwards towards the belly of the mother with the right hand, while with the left the cord is to be pulled downwards towards the anus. By this means the labour is ſaid to be frequently terminated in a few minutes. Sometimes, however, this operation ration muft be repeated at intervals for half an hour. When the obftacle to the delivery is by this means removed, and the head of the fœtus defcends and begins to dilate the external parts, the inftrument is to be taken away, and we are carefully to guard the Perinæum to prevent laceration.

If the face of the child is turned to the Pubis, it is to be firft pufhed afide as much as poffible, and as foon as by the operation of the lever the head begins to defcend, it will, we are affured, naturally fall into its proper fituation.

In cafes where the bafis of the fcull is entirely above the brim of the Pelvis, the large curve is to be ufed; and the fmall one when it has paffed that ftrait.

Among other caufes of laborious births our author mentions the oblique fituation of the uterus. This idea which was firft fuggefted by Daventer, has been fo often and fo ably refuted by fucceeding writers that we fhall not detain our readers upon the fubject, and the rather as M. Herbiniaux has brought no new argument in fupport of his doctrine.

Towards the end of the volume we meet with feveral cafes felected from a great number in which the author has employed the lever. In

 thefe, these, as well as in the other parts of his work he displays considerable knowledge of his subject, as well as zeal for the cause he has undertaken to defend. But after all it may be doubted whether the practitioners who have been instructed in the use of the forceps will be induced to relinquish them in favour of the lever. Both of the instruments are doubtless in some few cases useful; but either of them will require more practice, to become expert in handling them than almost any one will obtain, who does not frequently have recourse to them unnecessarily. Of the two however, the manual of the lever is certainly the most simple.

In our next number we mean to give an account of the second volume of this work, in which this author treats of the uterine polypus.

